# Antihyperglycemic Effect of *Rosa Damascena* is Mediated by PPAR.γ Gene Expression in Animal Model of Insulin Resistance

**Published:** 2017

**Authors:** Abbas Mohammadi, Hossein Fallah, Ahmad Gholamhosseinian

**Affiliations:** *Department of Clinical Biochemistry, Afzalipour School of Medicine, Kerman University of Medical Sciences, Kerman, Iran.*

**Keywords:** Insulin resistance, *Rosa damascene*, Adiponectin, Pioglitazone

## Abstract

Insulin resistance is a condition in which insulin signaling and action are impaired in insulin sensitive tissues and result in hyperglycemia, hyperlipidemia, and type 2 diabetes mellitus. Our previous studies have shown that *Rosa damascena* has antihyperglycemic effects on diabetic and normal rats. Therefore, we conducted a study to evaluate the effect of this medicinal plant on insulin sensitivity in rats. This study was performed on high fructose diet insulin resistant rats and pioglitazone, an insulin sensitizing drug, was used as a positive control. Insulin resistance was developed in animals by high fructose diet within six weeks. Then, *Rosa damascena* extract and pioglitazone were administered by gavage for two weeks and results were compared with two control groups. After treatment period, serum glucose, insulin, adiponectin, triglyceride, and cholesterol were assayed in fasting state. Plasma free fatty acid profile was analyzed by GC. Liver PPAR.γ and muscle GLUT.4 gene expressions were assessed by real time PCR and western blotting. Animals were treated with *rosa damascena* extract showed levels of insulin (42 ± 2.7 pmol/L). adiponectin (5.6±0.17 μg/mL). glucose (129±4.7 mg/dL). and triglyceride (75 ± 9 mg/dl) which were significantly improved as compared with control group insulin (137 ± 34 pmol/L), adiponectin (3.9±0.15 μg/mL). glucose (187±15 mg/dL). and triglycerides (217±18 mg/dL). PPARγ protein level was also significantly increased in Rosa damascene treated group. Our results demonstrated that *rosa damascena* extract has useful effects on insulin resistant animals and by increasing insulin sensitivity can be considered as a potential agent in control of diabetes.

## Introduction

Insulin resistance (IR) is a state that body tissues have less sensitivity to insulin and as a result, downstream metabolic pathways that are regulated by insulin are impaired and blood glucose rises (1). It is a prevalent metabolic disorder in industrialized countries and mainly associated with glucose intolerance, overt hyperglycemia, hyperinsulinemia and hypertension, a complex state that is referred to as metabolic syndrome (1, 2). 

Numerous factors have been identified to be associated with insulin resistance and type 2 diabetes mellitus. Obesity has a strong association with type 2 diabetes mellitus and this link is mediated by induction of insulin resistance (3). In obesity, adipocyte stress and hypertrophy and the resulting increase in proinflammatory adipocytokines (IL.6 and TNFα) contribute to the development of insulin resistance (4, 5). 

Adiponectin is the most abundant adipocytokine that is secreted from adipose tissue and has an inverse correlation with body fat. Plasma adiponectin is decreased in insulin resistance and type 2 diabetes mellitus (6). Adiponectin increases insulin sensitivity by decreasing hepatic gluconeogenesis and lipid storage (7).

Increased plasma free fatty acid is another key factor in the induction of insulin resistance. Free fatty acids affect numerous mechanisms such as activation of inflammatory pathways, alteration in phosphorylation of signaling molecules and synthesis of lipid intermediate (eg. diacylglycerol) (8-10). 

Thiazolidinedione’s (e.g. pioglitazone) are agonists of PPAR.γ and are used to sensitize the body to respond to insulin (11). Upon binding its ligand, PPAR.γ sits on Peroxisome Proliferator Response Elements on the DNA and changes the expression of target genes which ultimately result in increased insulin sensitivity (12). 

Discovering new agents for treatment of type 2 diabetes mellitus is highly demanded and plants are a potential useful source for this purpose. In our previous study we reported that *rosa- damascena*-flower-extract has an inhibitory effect on Alpha glucosidase and therefore decreases postprandial blood glucose level in normal and diabetic rats after administration of maltose (1 g/kg body weight( (13). However, in further studies (data not published) we also found that this extract inhibited the elevation of postprandial blood glucose after administration of Glucose, a sugar which is not a substrate for Alpha glucosidase We also found that the extract diminished the fasting blood glucose after administration for 15 days in diabetic rats (results not published). For this reason we designed this study to investigate the mechanism (s). Through which *rosa damascena* extract affects blood glucose level

## Experimental

*Animals*

Insulin resistant rats were used to study insulin secretion, insulin resistance, adiponectin and free fatty acid levels and Glucose Transporter 4 (GLUT.4) and Peroxisome proliferator activated receptor γ (PPAR.γ) gene expression.

*Plant extract*

Flowers of* Rosa damascena* were collected from Lalehzar, Keman province, Iran. Air dried flowers (300 g) were milled and extracted by maceration method in 1000 mL of methanol at room temperature for 48 h. After filtration, the extract was concentrated by a rotary evaporator at 40 °C and dried in a 40 °C oven. Dried extract was stored at -20 °C.

*Animal treatment*

Male Wistar rats weighing 270–280 g were obtained from the animal house of Kerman University of Medical Sciences. Rats were housed in cages (four animals per cage) at 22 ± 3 ◦C with 12 h of light. Rats were fed with standard chow and fresh tap water for 2 weeks. After 2 weeks, rats were divided randomly into 4 groups (n=8). Three groups were fed a 60% fructose diet and the fourth group as the healthy control group (HCG) was fed with standard diet (14). After 6 weeks, insulin resistance in fructose fed rats was confirmed by oral glucose tolerance test (OGTT) (results not shown). Animals were treated by *Rosa damascena* extract (100 mg/Kg) (13) and Pioglitazone (10 mg/Kg) (15). and were compared with two control groups: healthy control (fed on standard chow) and insulin resistant control (were fed a high fructose diet) 

Animals were treated by intragastric injection and treatment was continued for 2 weeks. Water and food intake were measured daily during this period. Body weight was also measured weekly throughout the 8 weeks of treatment.

**Table 1:** primers used in this study for real time PCR

**Accession number**	**PCR product**	**primers**	**Gene**
NM_012751	106	F: ACTGGCGCTTTCACTGAACT	GLUT.4
		R: CGAGGCCAAGGCTAGATTTTG	
NM_001145367	131	F: CATGCTTGTGAAGGATGCAAG	PPAR.γ
		R: TTCTGAAACCGACAGTACTGACAT	
NM_017008	138	F: TGGAGTCTACTGGCGTCTT	GAPDH
		R: TGTCATATTTCTCGTGGTTCA	

Cycle of threshold (CT) for each sample was determined. ΔCT was calculated using the following equation:

ΔCT = CT (target gene) – CT (endogenous reference gene (GAPDH))

Results were analyzed by 2^-ΔΔCT^ method.

To perform the real time PCR procedure, all suggestions made by the Qiagen in "Critical Factors for Successful Real Time PCR" were applied.

**Table 2:** Effect of *rosa* *damascena*on on body weight, water, food intake and other insulin resistance related parameters.

**parameter**	**groups**				**P** **-** **value** ^¥^
	HCG	Con	Pio	RDE	
weight in 6 week (g)	283±9	285±8	292±13	286±12	0.986
weight in 8 week (g)	285±9	307±8	294±16	275±9	0.042
Weight gain (g)	2±1.54^*^	22.25±11.49^#^	2.17±0.52^#^	-10.75±5.81^*^	<0.0001
Water intake (mL)	35±0.8^*^	47±2^#^	60±2^# *^	56±1.2^#^	0.151
Food intake (g)	21.6±0.7^*^	13.4±0.4^#^	13.5±0.3^#^	12.8±0.1^#^	0.978
Insulin (pmol/L)	50±4.8^*^	137±34^#^	40±2.7^*^	42±2.7^*^	<0.0001
Adiponectin (μg/mL)	2.9±0.16	3.9±0.15	5.6±0.4^# *^	5.6±0.17^ * #^	<0.0001
Glucose (mg/dL)	132±4^*^	187±15^#^	129±5.8^*^	129±4.7^*^	0.002
HOMA.IR	2.7±0.37^*^	9.7±2.1^#^	2.1±0.12^*^	2.2±0.18^*^	<0.0001
Cholesterol (mg/dL)	71±12.1	59±3.2	63±4.2	55±3.18	0.986
Triglyceride (mg/dL)	85±13^*^	217±18^#^	200±51^#^	75±9^ *^	<0.0001

HCG group was fed standard chow and the other three groups were fed a 60% fructose diet for 8 weeks. In Pio and RDE groups after the first 6 weeks, animals were treated by pioglitazone (10 mg/kg/day) or *rosa damascena*extract (100 mg/kg/day) for 2 weeks. Biochemical parameters were measured by an auto analyzer. Hormones were analysed by ELISA method. HOMA-IR was calculated as mentioned above. All data are presented as Mean ± SEM. (n=8)

HCG: healthy control group, Con: control, Pio: pioglitazone, RDE: *Rosa damascena *extract

**Table 3:** Effect of *rosa damascena*on on plasma free fatty acids profiles

**parameter**	**groups**				^*^ **P-value**
	HCG	Con	Pio	RDE	
myristic acid (μmol/L)	1.07±0.01	1.12±0.06	1.2±0.06	1.33±0.1	0.983
palmitic acid (μmol/L)	5.9±0.57	11.1±2.6	5.1±0.18	9.39±0.82	0.034
palmitoleic acid (μmol/L)	1.06±0.04	1.4±0.12	1.1±0.01	1.59±0.49	0.936
stearic acid (μmol/L)	1.3±0.12	2.7±0.12	2.06±0.11	2.2±0.23	0.837
oleic acid (μmol/L)	2.2±0.21	2.9±0.22	1.7±0.08	2.82±0.69	0.996
total free fatty acids (μmol/L)	12.53±1.95	22±2.7	15±2.3	21.82±0.94	0.999

HCG was fed a standard chow and three other groups were fed a 60% fructose diet for 8 weeks. In Pio and RDE groups, after the first 6 weeks, animals were treated by pioglitazone (10 mg/kg/day) or *rosa damascena *extract (100 mg/kg/day) for 2 weeks. FFAs were analyzed by gas chromatography as free fatty acid methyl esters after extraction from TLC plates. All data are presented as Mean± SEM. (n=8)

HCG: healthy control group, Con: control, Pio: pioglitazone, RDE:* Rosa damascene *extract

**Figure 1:**
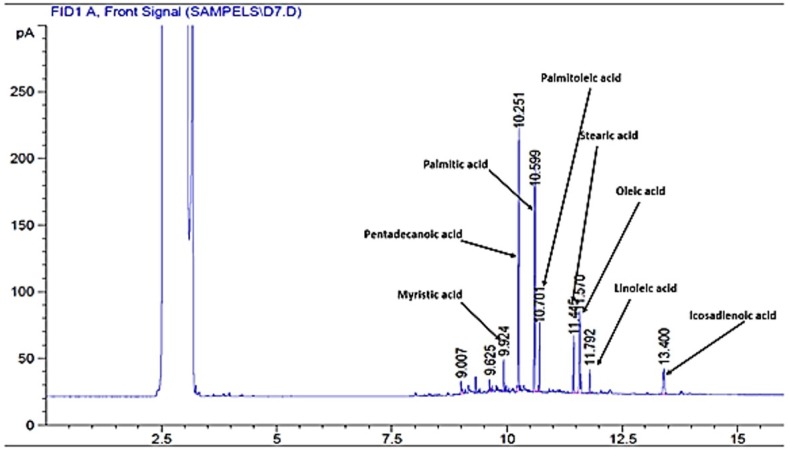
Chromatogram of plasma free fatty acids after administration of *rosa damascene*

**Figure 2:**
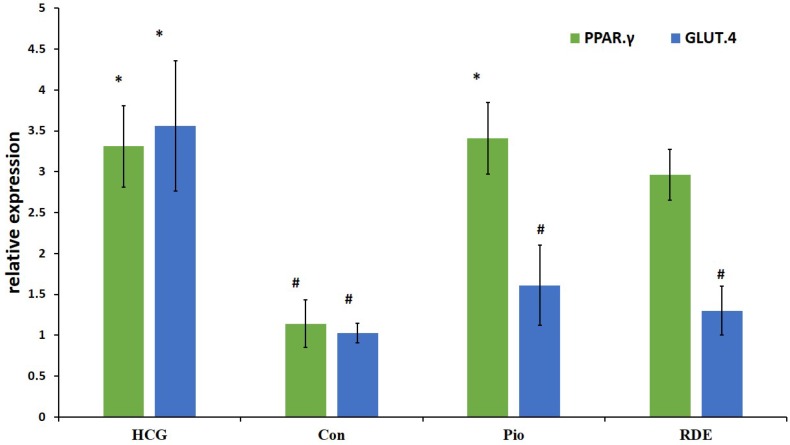
Effect of *rosa damascena* on mRNA levels of liver PPAR.γ and muscle GLUT.4

HCG was fed a standard chow and three other groups were fed a 60% fructose diet for 8 weeks. In Pio and RDE groups, after the first 6 weeks, animals were treated by pioglitazone (10 mg/kg/day) or *rosa damascena *extract (100 mg/kg/day) for 2 weeks. PPAR.γ and GLUT.4 mRNA levels in liver and muscle respectively, were quantified by real time PCR. All data are shown as Mean± SEM. (n=8)

HCG: healthy control group, Con: control, Pio: pioglitazone, RDE: *Rosa damascena *extract

* Significant difference with control group (P<0.05)

# Significant difference with HCG group (P<0.05

**Figure 3:**
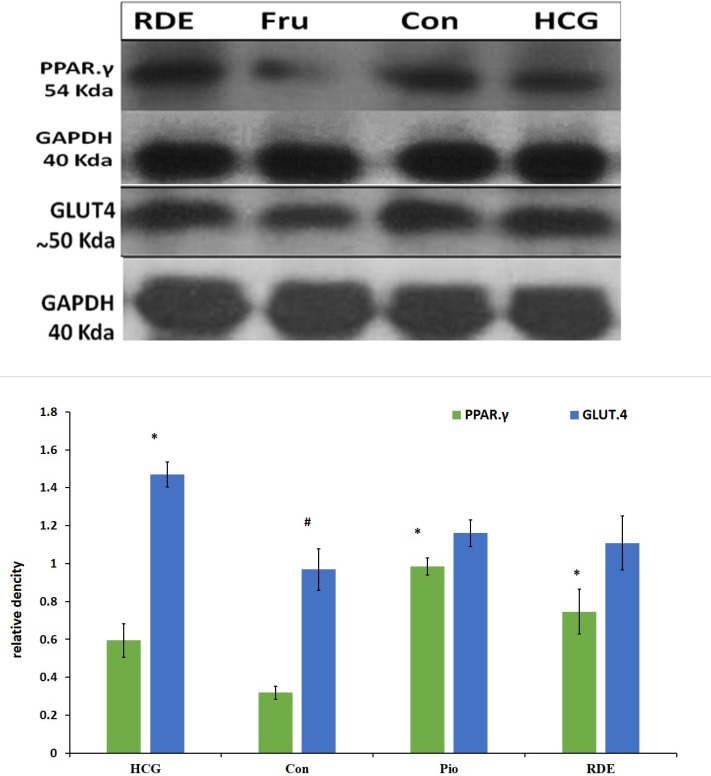
Effect of *rosa damascena* on protein level on liver PPAR.γ and muscle GLUT.4

HCG was fed a standard chow and three other groups were a fed 60% fructose diet for 8 weeks. In Pio and RDE groups after the first 6 weeks, animals were treated by pioglitazone (10 mg/kg/day) or *rosa damascena *extract (100 mg/kg/day) for 2 weeks. PPAR.γ and GLUT.4 protein levels in liver and muscle, respectively, were assayed by western blotting.

HCG: healthy control group, Con: control, Pio: pioglitazone, RDE:* Rosa damascena* extract

* Significant difference with control group (P<0.0001)

# Significant difference with HCG group (P<0.05)

*Blood and tissue collection*

Animals were fasted for 12 h. overnight, blood samples were collected from heart under ether anesthesia. Blood sample was divided into two vials with or without EDTA. Vials were centrifuged at 3000 rpm for 10 min at 4 °C and plasma (for FFA analysis) or serum (for other biochemical analyses) were separated immediately. 

Hind limb skeletal muscle and liver were excised. Tissue samples were immediately frozen in liquid nitrogen, and subsequently stored at −75 °C until required.

*Measurement of serum parameters*

Serum glucose, triglyceride, cholesterol and HDL-c concentrations were measured in an RA-1000 autoanalyser. Blood insulin and adiponectin levels were measured by ELISA method using a commercial assay kits (Mouse/Rat Adiponectin or insulin ELISA kit, USCN. China). Homeostasis Model Assessment of Insulin Resistance (HOMA-IR) was calculated using the equation [(insulin (µU/mL)×glucose (mmol/L))/22.5].

*Analysis of plasma free fatty acid profiles*

Plasma FFAs were extracted and analyzed by the method explained by Kangani *et al.* with slight modifications (16). Five hundred μL of plasma was mixed with 20 μL of pentadecanoic acid (1 mg/mL) as an internal standard. Lipids were extracted from plasma by a solvent which contained isopropanol–heptane–hydrochloric acid (1M) (40:10:1, v/v/v). FFAs were separated by Teen Layer Chromatography (TLC) on silica gel plates using a heptane–ether–acetic acid [60:40:3] solvent system. Lipids were visualized by iodine vapor on TLC plates. FFA bands were scrapped and free fatty acid methyl esters (FAME) were prepared by a reaction with BF3 in methanol (Sigma). FFA methyl esters were separated using an Agilent GC-7890A system equipped with a flame ionization detector and DB-225 capillary column (20m×0.1 mm I.D., 0.1μm film thickness, J&W GC columns, USA). The injection volume was 1μL in the split 30:1 injection mode.

*Real time PCR:*

Total RNA from the skeletal muscle (for GLUT.4 assay) and liver (for PPAR.γ assay) tissues was extracted with RNeasy mini kit (Qiagen) according to manufacturerʹs guidelines. The RNA concentration was determined by the ultraviolet (UV) light absorbance at 260 nm and 280 nm (ND-1000 Nanodrop). The quality of the RNA was confirmed by ethidium bromide staining of 18S and 28S ribosomal RNA bands after electrophoresis in a 2% agarose gel.

Then cDNA synthesis was performed by Quanti Tect Reverse Transcription Kit (Qiagen) according to the manufacturerʹs procedure. Relative Quantitative real time PCR was performed on a Qiagen Thermal Cycler (Rotor-Gene Q 5plex HRM System, Qiagen) using the corresponding QuantiFast SYBR Green PCR kit (Qiagen) according to manufacturerʹs protocol. The primers that were used in this study are shown in table 1.

*Western blotting*

Total protein was extracted from muscle or liver by homogenization of the tissue in the RIPA (Radio Immuno Precipitation Assay) buffer (Sigma, cat number: R0278). The homogenate was centrifuged at 14,000 rpm for 20 min and the supernatant which contain proteins was removed. Protein concentration was measured by Bradford method. Proteins were separated using SDS-PAGE by loading 120 μg/lane. Proteins were then transferred on a PVDF membrane. Non-specific binding sites were blocked by overnight incubating of membrane with 5% (usually a 5% solution is used) skim milk in TBST buffer at 4 °C. The membrane was then washed with TBST buffer 3 times (20 min each).

The membrane was incubated with appropriate polyclonal primary antibodies for PPAR.γ (ab27649, Rabbit polyclonal to PPAR gamma, Abcam) or GLUT.4 (ab33780, Rabbit polyclonal to GLUT4, Abcam) antibody in TBST buffer for 1 h. The membrane was then washed with TBST as described above, followed by incubation with anti-rabbit secondary antibody (Goat polyclonal HRP conjugated antibody to rabbit IgG, ab 6112, Abcam). For 1 h. at room temperature. After washing, membrane was incubated with substrate (western lightening plus ECL, Perkin-Elmer) for 1 min. Then in a dark room PVDF membrane was exposed to Hyblot film (Denvill) for 30 sec. Band densities were analyzed by image-J software.

*Statistical analysis*

All data are presented as mean±SEM. Statistical analysis was performed by analysis of variance (ANOVA) and Post-Hoc Tukey test; p-values of less than 0.05 were considered to be significant.

## Results

*Weight gain and water and food intake*

Weight gain that was calculated by subtraction of primary body weight from body weight at week 6 (before drug or extract administration) showed a significant difference between *Rosa damascena* Extract (RDE) and control groups (P<0.0001). There was no significant difference in water or food intake between RDE group and pioglitazone or control groups (results are shown in Table 2). Results show that *Rosa damascena* can induce weight loss in animals in spite of intake of high fructose diet. 

*Serum biochemical analysis*

*Roasa damascena* significantly decreased the levels of glucose (129±4.7 mg/dL), triglyceride (75 ± 9 mg/dL). Insulin (42 ± 2.7 pmol/L). HOMA.IR (2.2±0.18) and increased adiponectin in comparison with the control group with glucose (187±15 mg/dL), triglyceride (217 ± 18 mg/dL), insulin (137 ± 34 pmol/L). Adiponectin and HOMA.IR (9.7 ± 2.1). 

Serum adiponectin was significantly increased by *Rosa damascena* extract (5.6 ± 0.17 µg/mL) compared to the control (3.19 ± 0.15 µg/mL) and healthy control (2.9 ± 0.16 µg/mL) groups (results are shown in Table 2).

*Plasma free fatty acids*

*Rosa damascena* showed no significant effect on total free fatty acid level in comparison with the control or healthy control groups. However, pioglitazone significantly decreased the level of total free fatty acids, oleic acid, palmitic acid and palmitoleic acid as compared with the control and RDE groups (P<0.05) (results are presented in Figure 1 and Table 3).

*GLUT.4 and PPAR.γ gene expression*

Our results demonstrate that *rosa damascena* as well as pioglitazone significantly increase the mRNA level of PPAR.γ in liver. PPAR.γ protein also was significantly increased by *rosa damascena* and pioglitazone. However, neither *rosa damascena* nor pioglitazone didn’t have any effect on mRNA or protein levels of muscle GLUT.4 (figure 2).

This study showed that there is a significant difference in PPAR.γ protein levels between RDE and control groups. Also, pioglitazone significantly increased the level of PPAR.γ protein as compared with the control group. There was no significant difference in GLUT.4 protein level between RDE and control or pioglitazone groups (Figurer 3).

## Discussion

The goal of this study was to investigate the role of *rosa damascena* extract on insulin secretion and insulin action in animal model of insulin resistant. Our results showed that *rosa damascena* decreased blood glucose and insulin levels and thereupon HOMA-IR in insulin resistant rats. These results suggest that *rosa damascena* increases the insulin action and insulin sensitivity without increasing insulin secretion (17). Another possible mechanism is that *rosa damascena* has insulin like effect and through this decreases blood glucose level (18). 

Gholamhoseinian *et al.* reported that *rosa damascena* has an inhibitory effect on pancreatic lipase (19). however the lowering effect of this extract on TG in hryperlipidemic rabbits was not significant (20). However, in our study, *rosa damascena* extract, unlike pioglitazone, significantly decreased the level of blood TG in insulin resistant rats. ATPIII guideline recommends that in treatment of metabolic syndrome, lowering triglycerides should be considered (21). Therefore, RDE seems to be more suitable than pioglitazone for treatment of metabolic syndrome. 

The concentration of adiponectin was increased by administration of *rosa damascena* extract. Several factors, including insulin and Thiazolidinediones are associated with regulation of adiponectin secretion (22, 23). Adiponectin increases insulin sensitivity by suppression of hepatic gluconeogenesis and stimulation of fatty acid oxidation in muscles (24). These findings are sufficient to conclude that antihyperglycemic effect of RDE may be due to stimulation of adiponectin production. 

At the molecular level, RDE increased the mRNA and protein of PPAR.γ in the liver, however RDE had no effect on GLUT.4 protein and mRNA in the striated muscle. PPAR-γ agonists exert their antidiabetic effect by PPAR-γ activation to increase the sensitivity of insulin receptors (25). Thiazolidinediones significantly increase insulin sensitivity and adiponectin concentration in diabetic patients (25, 26). Yadav *et al*. showed that PPAR.γ mRNA increased in liver of insulin resistant rats that were treated with rosiglitazone (27). PPAR.γ protein decreased in fructose fed rats and increased after treatment with pioglitazone and RDE. Similar to the role of PPAR.γ in improvement of insulin resistance, it is likely that increased PPAR-γ protein is another mechanism through which RDE applies its antihyperglycemic effect.

Agonists of PPAR.γ increase the expression and translocation of GLUT4 in adipose tissue, and increase the catabolism of glucose in the liver along with decreasing hepatic glucose output (28). We don’t sow study any difference between RDE and control group in GLUT.4 levels. Also pioglitazone didn’t have any effect on GLUT.4 protein and mRNA. In this study we evaluated total GLUT.4 in striated muscle cells. It is possible that translocation of GLUT.4 to the cell membrane after treatment with RDE and pioglitazone is increased despite the unchanged total GLUT.4 protein between groups (29). 

In diabetes, the levels of free fatty acids are increased and there is a reversed correlation between plasma FFAs and insulin sensitivity in muscles (30). Although in our study there was a significant difference in serum total free fatty acids between healthy control and control groups which supports the relationship between free fatty acids and insulin resistance, total FFA or FFA fractions did not significantly change with RDE in insulin resistant rats despite increase in insulin sensitivity by RDE. Our results showed that pioglitazone (a PPAR.γ agonist) decreased the level of free fatty acids. PPAR.γ agonists enhance insulin sensitivity in adipose tissue and reduce serum FFA levels by increasing their uptake and storage in adipose tissues (31, 32). However, in our study, FFA level was not significantly changed by *rosa damascena* while PPAR.γ protein was increased. We only evaluated PPAR.γ gene expression in the liver to examine its effect on hepatic insulin resistance and it is possible that PPAR.γ gene expression in adipocytes does not increase.

## Conclusion

The findings of this study showed that *rosa damascene *water extract can decrease blood glucose and triglyceride levels in insulin resistant rats. This antihyperglycemic effect was probably exerted at least by three mechanisms, including an increase in insulin action or a direct insulin-like effect, increase in adiponectin and increased PPAR.γ protein expression. Therefore, rosa damascene, because of its antihyperglycemic and antihyperlipidemic effects, can be a good candidate to control diabetes 
